# Robot-Assisted Cardiovascular Interventions

**DOI:** 10.1016/j.jscai.2025.102568

**Published:** 2025-03-18

**Authors:** Jon C. George, Vincent Varghese, Ryan D. Madder

**Affiliations:** a Division of Cardiology, Thomas Jefferson University Hospital, Philadelphia, Pennsylvania; b Division of Cardiology, Deborah Heart and Lung Center, Browns Mills, New Jersey; c Frederik Meijer Heart & Vascular Institute, Corewell Health West, Grand Rapids, Michigan

**Keywords:** robotic endovascular, robotic percutaneous coronary intervention, telerobotic, telestent

## Abstract

Innovation has been the cornerstone of progress in the field of percutaneous coronary intervention (PCI) since its inception. Refinements in procedural technique and interventional tools have improved patient outcomes and overall safety. Despite this progress, however, the health risks posed to operators and staff remain undeniably high. Robotic PCI (R-PCI) offers a new era in coronary revascularization poised to address this dilemma. To date, R-PCI procedures have been widely performed in clinical practice for over a decade and multiple novel endovascular robotic systems are currently under development. This review serves as an up-to-date understanding of R-PCI, focusing on the origins, clinical evidence, current state, and future targets of robotic therapy.

## Introduction

Innovation has been the cornerstone of progress in the field of percutaneous coronary intervention (PCI) since its inception. Refinements in procedural technique and interventional tools have improved patient outcomes and overall safety. Despite this progress, however, the health risks posed to operators and staff remain undeniably high. Ionizing radiation exposure increases the risk of cancer and cataract formation, whereas the donning of lead aprons, to protect from radiation, is associated with debilitating orthopedic injury affecting the spine, hips, and knees. Robotic PCI (R-PCI) offers a new era in coronary revascularization poised to address this dilemma. To date, R-PCI procedures have been widely performed in clinical practice for over a decade. Multiple novel endovascular robotic systems are currently under development and hold the potential to help disseminate interventional expertise through telerobotic applications. This review serves as an up-to-date understanding of R-PCI, focusing on the origins, clinical evidence, current state, and future targets of robotic therapy.

## Origins of R-PCI

On September 16, 1977, the first percutaneous transluminal coronary angioplasty was performed by Dr Andreas Gruentzig in Zurich, Switzerland.^1^ The right common femoral artery was accessed with Dr Gruentzig standing to the right of the patient, next to a fluoroscopic imaging system. After decades of technological evolution, the basics of a cardiac catheterization procedure, wherein the physician stands adjacent to the patient and is exposed to scatter radiation, have largely remained unchanged. The majority of interventional physicians wear lead garments to protect themselves against ionizing radiation; however, supporting the weight of lead aprons comes at the cost of increased orthopedic complications.^2^ The most common orthopedic injuries reported are lumbar and cervical spine issues, as well as, hip, knee, and ankle joint problems.^3^^,^^4^ Radiation protection has seen a renaissance of interest over the past several years with multiple companies devising solutions as alternatives to wearing lead, such as floating lead aprons, or enhanced shield protection around the radiation source.^5^, ^6^, ^7^ Still, the uptake of these technologies has been measured due to the financial investment needed and unfamiliarity to the general interventionalist. The dangers of ionizing radiation exposure are well known with reports demonstrating early cataract formation specifically targeting the posterior subcapsular region of the lens.^8^^,^^9^ Over a lifetime of procedures, there is a 6-fold increased risk compared to the general population.^10^ Even more concerning is the possible increased risk of left-sided brain malignancies for interventional operators. Several small studies have demonstrated the cumulative effects of radiation may lead to a 2- to 3-fold higher risk of developing head and neck cancers.^11^, ^12^, ^13^ Ultimately, the risks faced by interventional cardiologists led to the development of robotic systems for performing PCI in order to address these occupational hazards.

## First- and second-generation R-PCI systems

The first R-PCI was performed with a prototype in 2004^14^ with a commercially available platform (CorPath 200, Corindus) receiving FDA approval in 2012. This first-generation R-PCI device was capable of manipulating coronary equipment with an operator seated in a control cockpit.^15^ Coronary guide wires and rapid exchange balloons/stents were manually inserted into the robotic arm, while joystick controls were used to advance and retract equipment under fluoroscopic guidance (Central Illustration).^16^ The catheterization laboratory staff could distance themselves from the radiation source and patient while the robotic system was in use, and apply standard radiation protection practices to lower overall exposure. Although novel in its design, several limitations of the first-generation robotic system existed, such as the need for a second operator to manually insert and remove equipment, the lack of ability to robotically manipulate a guide catheter, and the inability to handle more than 1 wire or 1 balloon/stent at the same time.

**Central Illustration:**
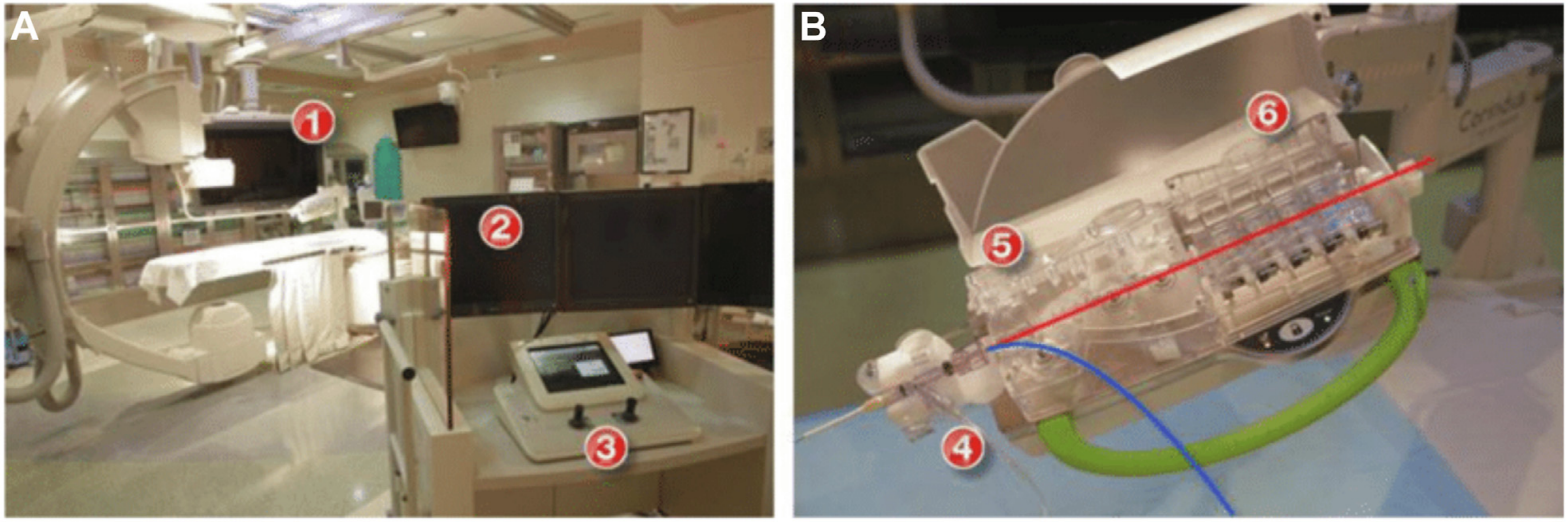
**Thefirst-generationvascular robotic system.** Panel A: Robotic percutaneous coronary intervention (PCI) platform including robotic arm and cassette (1); interventional cockpit (2); and control console (3). Panel B: Robotic cassette demonstrating placement for the guide wire (red) and the balloon/stent (blue) within the driver for rapid-exchange catheters (4); driver for 0.014′′ guide wires (5); and mechanical torque system (6).^16^

A second-generation R-PCI device (CorPath GRX, Corindus), approved by the FDA in 2016, intended to address some of these shortcomings.^17^ This second-generation device integrated robotically-directed guide catheter movement, such as advancement, retraction, and rotation. Guide wire manipulation was also enhanced with automation technology to replicate common wire maneuvers performed by operators, such as the “wiggle, spin, and rotate on retract” features.^18^ The notable limitations of the second-generation device included the inability to robotically support the use of over-the-wire catheters and devices, embolic protection devices, rotational/orbital atherectomy, most intracoronary imaging devices, and being unable to simultaneously robotically manipulate 2 wires or 2 balloons/stents at the same time.^19^ Although these first- and second-generation robotic systems are no longer in clinical use, it is important to note that the majority of clinical data supporting the safety and efficacy of both R-PCI and robotic peripheral interventions, summarized in the following sections, were generated using these devices over a time span of nearly a decade.

## Clinical evidence for R-PCI

### R-PCI in low to moderate lesion complexity

The PRECISE (Percutaneous Robotically Enhanced Coronary Intervention) study was the first extensive multicenter investigation assessing the safety and effectiveness of R-PCI.^15^ A total of 164 patients with documented obstructive coronary artery disease and signs of myocardial ischemia were enrolled, with R-PCI performed using the first-generation robotic system. Key inclusion criteria were a de novo stenosis of at least 50% in diameter, a maximum lesion length of 24 mm, and a reference vessel diameter between 2.5 to 4.0 mm, suitable for a single stent placement. Major exclusion criteria included prior stenting within 5.0 mm of the target lesion, anticipated atherectomy, presence of intraluminal thrombus, significant tortuosity or calcification, chronic total occlusion (CTO), ostial lesions, bifurcation involvement, or lesions in an unprotected left main coronary artery. All procedures were conducted via femoral access, achieving procedural success without the need for manual intervention in 98.8% of cases. The 2 instances requiring manual conversion were due to challenges in stent delivery, which also complicated the manual procedures, necessitating a transition to a more supportive guide catheter. Four cases of peri-procedural myocardial infarction, defined by a creatine kinase myocardial band enzyme increase greater than 3 times the upper limit of normal, occurred, but these were clinically inconsequential. No other significant adverse clinical outcomes were reported. The average procedure time using the robotic system was 24.4 ± 14.1 minutes, with a mean fluoroscopy duration of 11.1 ± 6.2 minutes. These findings, which were the first to support the safety and efficacy of R-PCI for treating coronary lesions of low to moderate complexity, were soon followed by data from a multicenter postmarket registry showing the safety and success rate of performing R-PCI from the radial approach.^20^ This study reported technical success rates of 93.7% with radial access and 85.7% with femoral access, with no major adverse cardiovascular events noted.

### R-PCI in complex lesions

The PRECISION GRX registry reported data on 980 patients undergoing R-PCI with the second-generation robotic system and found clinical success and technical success rates of 97.8% and 86.5%, respectively.^21^ In the PRECISION GRX registry, 68.8% of lesions were type B2/C thereby suggesting R-PCI can be performed in more complex lesion subsets.^21^ Additionally, early case reports of R-PCI in more complex PCI procedures (multivessel disease, bypass grafts, myocardial infarction, unprotected left main, hemodynamic support cases, laser atherectomies, and CTO revascularization) were published.^22^, ^23^, ^24^, ^25^ The Complex Robotically Assisted Percutaneous Coronary Intervention (CORA-PCI) study was a nonrandomized single-center comparison of R-PCI versus manual PCI in the treatment of complex coronary artery disease.^26^ Technical success was noted to be 81.5% in the R-PCI arm with manual assistance required in 11.1% and manual conversion in 7.4%. In-hospital major adverse cardiovascular events were low in both arms with comparable clinical success. Although stent use and fluoroscopy times were similar as well, the total procedure time was higher in the R-PCI group due to the robotic drive set-up time. The significance of the Complex Robotically Assisted Percutaneous Coronary Intervention study was that it provided additional evidence that R-PCI can be successfully performed even among more complex lesion subsets. In a study evaluating longer-term safety and efficacy outcomes with R-PCI, no significant differences were reported between R-PCI and manual PCI at 6- and 12-month follow-ups.^25^ Introduction of R-PCI for complex lesion subsets early in the learning curve of starting an R-PCI program has been described.^27^

### R-PCI in CTO

Although R-PCI was initially avoided in CTO lesions owing to the incompatibility of first- and second-generation robotic devices with over-the-wire systems, an early report of the feasibility of R-PCI in CTO lesions was eventually published.^28^ A hybrid approach to R-PCI in CTO has since been studied, in which crossing of the CTO was performed manually followed by ballooning and stenting performed robotically.^29^ This hybrid approach allows the operator to perform the latter portion of the CTO PCI from the robotic cockpit, thereby eliminating radiation exposure, facilitating the removal of lead aprons, and allowing the operator to be seated during the final stages of these conventionally longer and more complex cases. In an observational study performed at 2 centers, this hybrid R-PCI approach to treating CTO lesions had a 98% success rate.^29^ Furthermore, rates of major adverse events in the R-PCI group were not significantly different than those of a manual control group.

### Robotic peripheral interventions

**Figure 1:**
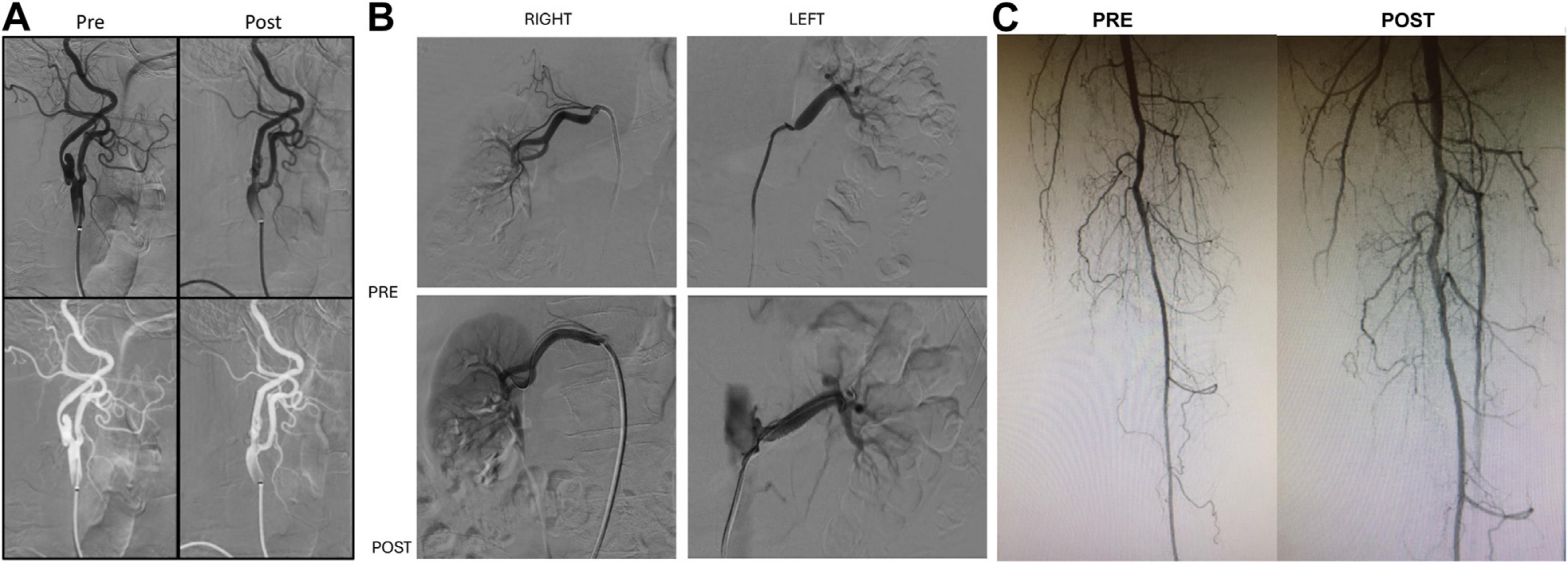
**Robotic carotid and renal interventions.** The first panel of images (A) demonstrates the first robotic carotid revascularization performed with preintervention images on the left and postintervention images on the right.^33^ The second panel of images (B) demonstrates successful bilateral renal revascularization performed robotically with preintervention images on the top bar and postintervention images on the bottom bar.^34^ The third panel of images (C) demonstrates successful left anterior tibial artery revascularization performed robotically with a preintervention image on the left and a postintervention image on the right.

Reports of using the first-generation robotic system to perform endovascular interventions began to emerge several years after R-PCI was established.^30^ The safety and feasibility of peripheral robotic interventions were demonstrated in 2016 in a prospective single-arm observational study of 20 patients with symptomatic femoropopliteal disease.^31^ In this study, the technical and procedural success of robotic peripheral intervention using the first-generation robotic system was 100% and no adverse events occurred. An additional study confirmed the safety and efficacy of robotic peripheral interventions and demonstrated the use of the robotic system to be associated with a marked reduction in radiation exposure to the physician operator.^32^ The use of robotic systems for other endovascular applications, including carotid, renal, and peripheral interventions (Figure 1),^33^^,^^34^ have been successfully demonstrated.^33^^,^^34^

A summary of clinical studies demonstrating the safety and efficacy of robotic interventions is listed in Table 1.^15^^,^^17^^,^^21^^,^^26^^,^^32^^,^^35^

**Table 1:** Clinical studies evaluating the safety and feasibility of robotic interventions.

Study/Reference, year	Robotic system	Patients	Technical success	Clinical success	Major adverse events
PRECISE,^15^ 2013	CorPath 200	164	98.8%	97.6%	2.4%
RAPID,^32^ 2020	CorPath 200	20	100%	100%	0%
CORA-PCI,^26^ 2017	CorPath 200	108	81.5%	99.1%	0.9%
Smitson et al,^17^ 2018	CorPath GRX	40	90.0%	97.5%	0%
PRECISION GRX,^21^ 2021	CorPath GRX	980	86.5%	97.8%	0%
R-EVOLUTION,^35^ 2023	R-One	62	95.2%	100%	0%

CORA-PCI, COmplex Robotically Assisted Percutaneous Coronary Intervention; PRECISE, Percutaneous Robotically Enhanced Coronary Intervention Study; RAPID, robotic-assisted peripheral intervention for peripheral artery disease.

## Advantages of robotic interventions

Although a learning curve for competency with R-PCI exists, 1 large multicenter study demonstrated that proceduralists can gain proficiency after their first 3 cases.^36^ Moreover, a single-center study showed success in reducing contrast volume, procedural times, and fluoroscopy times after 50 R-PCI cases.^37^ Additional potential benefits of R-PCI include a significant reduction in operator and staff radiation exposure, fewer orthopedic issues because wearing lead is not necessary within the control cockpit, and accurate stent placement mitigating the chance of geographic miss.^15^^,^^36^^,^^38^ A secondary effect of implementing an R-PCI program has been suggested to include team building and enhanced staff satisfaction,^27^ but these secondary effects have not been adequately studied.

A major benefit of R-PCI is the significant reduction in procedural radiation exposure to the operator. The PRECISE study demonstrated a 95.2% reduction in radiation exposure to the primary operator with R-PCI from within the radiation-shielded cockpit compared to the traditional tableside position.^15^ Furthermore, median radiation exposure to the operator’s head during R-PCI was noted to be significantly lower when compared with manual PCI with traditional lead or suspended lead systems.^39^ Additionally, 1 retrospective study reported less radiation exposure to the R-PCI–treated patient in comparison to manual PCI patients, which was attributed to being able to perform R-PCI cases with the procedure table at heights conventionally not utilized in manual cases.^40^

Another benefit of R-PCI is the impact it bestows on potential orthopedic injuries while allowing a seated position in comparison to a prolonged standing position while wearing lead-lined garments. Although there are no data to support this speculative benefit, the ability to perform long procedures while seated in ergonomically designed stations likely reduces the incidence of such orthopedic and musculoskeletal injuries.

Robotic PCI also allows for precise lesion length measurements and precise movement of devices in millimeter increments, thereby potentially reducing the incidence of longitudinal geographic miss and appropriate stent sizing. The PRECISE study confirmed the reduction of longitudinal geographic miss with R-PCI when compared to manual PCI,^41^ while other data demonstrated that in 8.3% of cases, R-PCI reduced the use of additional stents.^42^

## Future and emerging technologies

**Figure 2:**
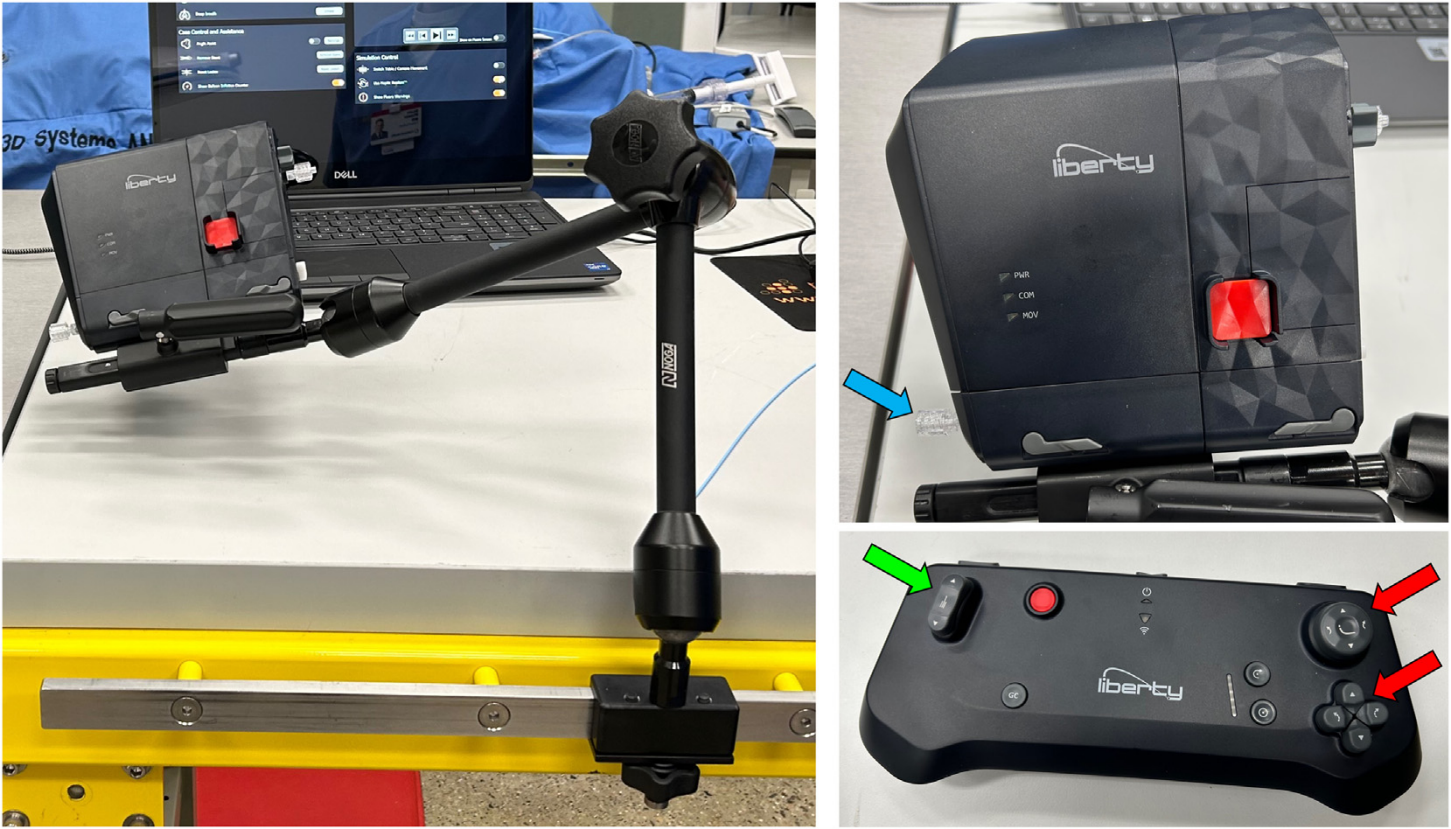
**Miniaturized robotic system.** The miniaturized disposable robot (upper right) attaches to a bedside articulated arm mounted on the procedure table (left) and is controlled by a wireless handheld controller (lower right). The location for the attachment of a catheter to the robot is shown (blue arrow). The handheld controller has a joystick and buttons for wire manipulation (red arrows) and a button for balloon/stent manipulation (green arrow).^43^

**Figure 3:**
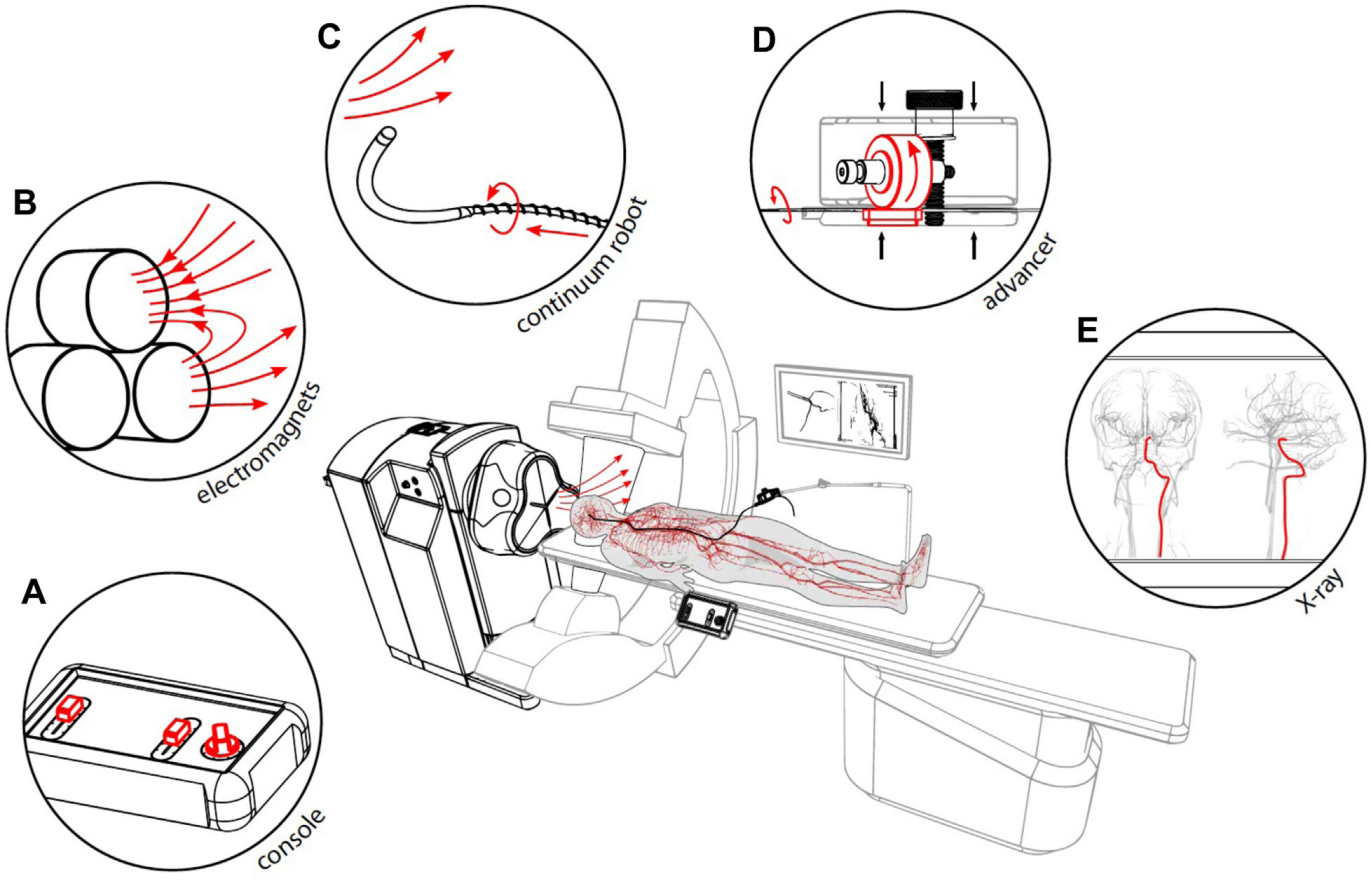
**Next-generation electromagnetic robotic system.** Shown is a schematic of an emerging next-generation electromagnetic robotic system. (A) The control console takes user inputs to control the magnetic field direction and device insertion speed. (B) The electromagnets generate the external magnetic field which is projected onto the patient and is used to steer the magnetic devices inside the patient. (C) The helical magnetic endovascular device. (D) The motorized advancer unit pushes and rotates the helical magnetic devices. (E) The x-ray image provides visual feedback of the vasculature and the magnetic device. A prototype of this system was recently utilized to perform a preclinical study of trans-Atlantic telerobotic coronary angiography between the US and Europe.^44^^,^^45^

In 2023, the first- and second-generation robotic systems were removed from the market and are no longer available for clinical use. With this development, the first era of robotics in the catheterization laboratory concluded, and in its wake, several novel endovascular robotic systems, some of which are summarized in Table 2, are now either in clinical use or under development. These next-generation robotic systems, which will form the basis of the second era of catheterization laboratory robotics, will enable the performance of R-PCI, peripheral interventions, and neurologic interventions, achieve high levels of technical and procedural success, and provide radiation protection for the physician operator similar to the previously available first- and second-generation robotic systems. These emerging robotic systems will also likely have increased functionality, capabilities to support a greater variety of adjunctive endovascular devices, and other novel attributes. For example, 1 of the robotic systems under development will enable haptic feedback, thereby providing the physician with sensory information regarding the forces acting on an endovascular device as it is being manipulated. Another novel approach is the emergence of a miniaturized endovascular robot (Figure 2)^43^ that is disposable and intended for single use, thus eliminating the requirement of a hospital to make a large capital purchase characteristic of most other robotic systems in order to develop a robotic program in their catheterization laboratory.^43^ Another endovascular robotic system currently under development will project an electromagnetic field onto the patient (Figure 3),^44^^,^^45^ and by manipulating the vector of the magnetic field the controlling physician can alter the tip shape of a magnetized guide wire in real-time.^44^

**Table 2:** Novel cardiovascular robotic systems in clinical use or development.

Robotic Company	Clinical use	Stage of development	Special features
Robocath	Endovascular	In clinical use	In clinical use for PCI—Europe and China
Microbot Medical	Endovascular	IDE trial completed for peripheral intervention in the US	Wireless, disposable, and intended for single use
Nanoflex Robotics	Endovascular	Preclinical	Utilizes electromagnetic field for device navigation
Sentante	Endovascular	Initial clinical trial enrolling in Europe	Provides haptic feedback
Telos Health	Endovascular	Preclinical	Focusing on telerobotic stroke treatment
XCath	Endovascular	Preclinical	Developing electrically actuated steerable guide wire
ROSES	Endovascular	First-in-human study completed	Focused initially on R-PCI

PCI, percutaneous coronary intervention; R-PCI, robotic percutaneous coronary intervention.

Of the next-generation robotic systems, only 1 is currently available for clinical use in Europe (R-One, Robocath) and none are yet available in the United States. The safety and efficacy of the R-One system was recently evaluated among 62 patients undergoing R-PCI.^35^ Using the R-One system, R-PCI was performed with a technical success rate of 95.2%, manual conversion in only 3 cases, and no major safety events during the procedure or at 30 days. Furthermore, the use of this next-generation robotic system was associated with significant reductions in radiation exposure to the physician operator. Additional investigation is underway to evaluate the real-world performance and safety of the R-One system in R-PCI,^46^ and another emerging robotic device, the ROSES robotic system, recently completed its first-in-human R-PCI cases.^47^ At least 2 other next-generation endovascular robotic systems are being studied in the clinical setting for performance of peripheral vascular interventions, with 1 trial enrolling in Europe using the Sentante robotic system capable of providing the operator with haptic feedback^48^ and the other having recently completed enrollment in a trial in the United States with the miniaturized disposable robotic system.^49^

## Telerobotic endovascular interventions

Importantly, it is likely that all of the novel endovascular robotic systems currently being developed will be capable of use in telerobotic procedures (Table 2). The impetus to develop telerobotic endovascular capabilities stems from 2 ongoing issues of increasing public health importance. First, there are marked geographic disparities in patient access to endovascular procedures, as exemplified by recent evidence showing that patients with myocardial infarction in the US are significantly less likely to receive coronary angiography and PCI if they present to a rural compared to an urban hospital.^50^ Similar rural-urban disparities exist among patients with stroke, as many stroke patients, although considered eligible for endovascular thrombectomy, do not have access to a hospital with thrombectomy capabilities.^50^^,^^51^ Second, a shortage of cardiologists and other specialized physicians having the expertise to perform endovascular procedures is predicted to impact the US over the next decade.^52^ This physician shortage has the potential to exacerbate limited access to endovascular procedures in rural markets. If used to disseminate procedural expertise, telerobotic technology is 1 solution that could be applied to address these disparities.

The concept of using a robotic system to perform telerobotic surgery is not new, as evidenced by the so-called Lindberg Operation in 2001 in which a surgeon in New York successfully performed a telerobotic cholecystectomy on a patient in Strasbourg, France.^53^ The Lindberg Operation spawned 2 decades of research into telerobotic surgery, including several contemporary telerobotic surgeries in humans^54^^,^^55^ and some higher-profile developments such as the performance of telerobotic surgery research on the International Space Station.^56^

**Figure 4:**
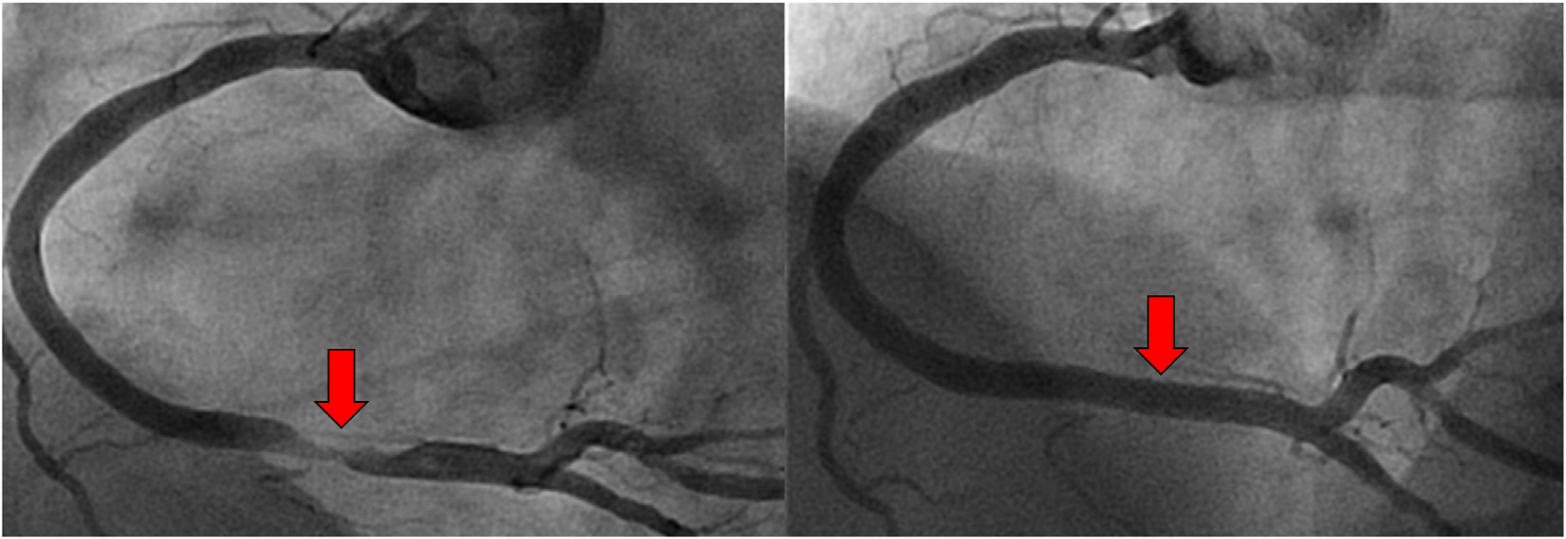
**Myocardial infarction treated with robotic percutaneous coronary intervention (R-PCI).** Shown is the pre-PCI (left) and post-PCI (right) angiogram of the first patient enrolled in the REMOTE PCI study^56^ on December 1, 2014. The location of the culprit lesion responsible for the myocardial infarction is shown by the red arrow. This robotic percutaneous coronary intervention (PCI) was successfully performed by a physician who was not present in the procedure room housing the patient. Using robotic controls, the physician performed the PCI while located in a separate closed room at a distance of approximately 55 feet from the patient. To our knowledge, this study was the first to evaluate the feasibility of telerobotic PCI.

Similar to research in telerobotic surgery, there is a growing body of research aimed at developing telerobotic PCI, which began in 2014 with an initial study demonstrating the feasibility of a physician performing R-PCI from outside the procedure room housing the patient.^57^ This initial study, wherein the physician performing PCI was 55 feet away from the patient (Figure 4),^56^ was subsequently followed by ex vivo studies successfully demonstrating telerobotic PCI performed over longer distances in simulators^58^^,^^59^ and then in in vivo studies in animals.^58^^,^^60^ These preclinical studies served as the foundation for the successful performance of telerobotic PCI in 5 humans over a distance of 20 miles in India in 2018.^61^ Additional demonstrations of telerobotic PCI in humans over long distances have been conducted more recently. The development of other telerobotic endovascular procedures is also underway, including telerobotic peripheral arterial interventions and telerobotic neurovascular procedures.^45^^,^^62^

As telerobotic endovascular procedures continue to develop, geographic distance may not be a significant barrier due to the emergence of ultrafast network connectivity, including fifth-generation wireless technology. Indeed, telerobotic studies over fifth-generation wireless networks have demonstrated their ability to support ultralong distance telerobotic interventions in a reliable manner, including preclinical studies of telerobotic PCI between Boston and San Francisco^59^ and between the US and Europe.^63^ In the latter example, trans-Atlantic control of an endovascular robotic system was successfully demonstrated between the United States and Switzerland.^63^

Prior to the entry of telerobotic endovascular interventions into routine clinical care in the US, additional studies are needed to address some unresolved limitations of this novel approach to delivering cardiovascular care. Among these, the need to establish redundant network connectivity is important to ensure that a telerobotic procedure could continue without interruption in the event that 1 type of network connection was lost midprocedure. Data security remains a major concern and also requires additional study. There is also uncertainty regarding the level of training and expertise of medical personnel attending to the patient during a telerobotic procedure to ensure that complications can be managed when they occur. Finally, several legal and regulatory hurdles will need to be overcome to enable telerobotic procedures across state lines.

## Conclusion

Robotic coronary and endovascular interventions represent an exciting development in the field of interventional cardiology and endovascular medicine. There is a clear benefit to the operator in reducing radiation exposure and orthopedic injuries while optimizing technical success with the reduction of longitudinal geographic miss and decreasing stent use. Telerobotic interventions which help with the ability to perform procedures remotely add a new dimension of options with widespread applications that remain to be explored further. Technological advances and feasibility studies will continue to evolve in this space in the future.
